# The Role of Practitioner- and User-Set Goals in Engagement and Psychological Distress Among Kooth Digital Health Users: Retrospective Analysis

**DOI:** 10.2196/70818

**Published:** 2025-12-15

**Authors:** Jacqlyn Yourell, Jennifer Huberty, Terry Hanley, Louisa Salhi

**Affiliations:** 1 Fit Minded Inc. Phoenix, AZ United States; 2 University of Manchester Manchester United Kingdom; 3 Kooth Digital Health London United Kingdom

**Keywords:** youth, young adults, mental health, psychological distress, digital mental health, goal setting, practitioner

## Abstract

**Background:**

Youth and young adult mental health concerns are rising globally, with digital mental health platforms offering a promising solution for accessible support. Among the various features these platforms provide, goal setting and achievement have been shown to positively influence behavior change and mental health outcomes. However, there is limited understanding of how user-set goals compare to those set collaboratively with a practitioner regarding their impact on user engagement and mental health outcomes in digital mental health platforms.

**Objective:**

The purpose of this study was to examine the relationship between various goal-related variables (eg, the number of goals created and progress in user-set and practitioner-set goals) and user engagement as well as mental health (ie, psychological distress) on a free digital mental health platform. A secondary exploratory aim was to assess how different user-presenting issues were associated with platform engagement.

**Methods:**

We leveraged secondary data from a free, web-based mental health platform for youth aged 10 to 25 years in the United Kingdom that offers goal-setting features, emotional journaling, peer support, asynchronous chat with practitioners, and various self-guided well-being activities. Data included in the analyses were from youth and young adults (mean age 15.84 years, SD 2.88; 522/691, 75.5% female) who engaged with the goal-setting feature and completed both pre- and postengagement psychological distress measures between January 2020 and December 2023. We examined the relationship between user-set goals and practitioner-set goals on user engagement and psychological distress via linear regressions. The impact of different user-presenting issues on engagement was also explored via linear regression.

**Results:**

The number of practitioner-set goals created was positively associated with platform engagement (β=.16; *P*<.001), whereas the number of self-set goals and goal progress, whether self or practitioner set, were not. Progress on practitioner-set goals was significantly associated with reduced psychological distress (β=–.27; *P*<.001), while progress on self-set goals showed no significant association (*P*=.16). Physical health–related and school-related presenting issues were the strongest predictors of increased platform engagement (β=.21; *P*<.001 and β=.17; *P*<.001, respectively).

**Conclusions:**

These findings underscore the importance of collaborative goal setting in improving mental health outcomes for youth and young adults on digital mental health platforms. By highlighting the role of guided support and goal progression, this study enhances our understanding of how digital mental health platforms can better support young people’s mental health and well-being. This paper also highlights how digital mental health platforms can serve as a valuable resource for addressing a wide range of mental health needs.

## Introduction

Youth mental health concerns are continuing to grow globally, with 1 in 7 adolescents aged 10 to 19 years experiencing a mental disorder. Suicide is the third leading cause of death among those aged 15 to 29 years [1]. In the United Kingdom, more than 1 in 5 children and young adults (aged 8-25 y) experience a mental health disorder [2]. The growing need for accessible mental health support has fueled the popularity of digital mental health platforms, which offer scalable, real-time support that can be accessed from almost anywhere, anytime [3,4]. As the demand and use of digital mental health platforms among youth rise, understanding the factors that drive engagement and positive mental health outcomes is imperative.

User engagement with digital mental health platforms, even those that are free, can be significantly low. For example, a review of more than 93 free digital mental health apps with at least 10,000 downloads revealed that more than 90% of the users discontinued use within 30 days of installing the app [5]. However, this does not account for “episodic engagement,” in which users engage with the app as needed rather than daily [6]. The review also excluded apps with therapist-based components to focus on unguided interventions. More research is needed to identify the factors influencing user engagement in digital mental health platforms with guided components, particularly among adolescents and young adults, and over longer periods (>30 d).

Interestingly, the review found that self-guided apps did not show improvements for adolescent and young adult mental health [5]. However, some have reported that therapist-based or guided interventions may lead to better engagement and mental health outcomes, particularly for those experiencing greater distress, as they provide users with structured support and accountability [7,8]. Research suggests that professional guidance in digital mental health interventions is more effective at improving mental health outcomes in adults (aged >18 y) compared to unguided interventions [9]. Further research is needed to identify aspects of self-guided and practitioner-guided strategies that influence mental health outcomes among adolescents and young adults in digital mental health platforms.

Goal setting is an effective strategy for behavior change. A systematic review and meta-analysis of 155 studies found that interventions that involve goal setting can improve a range of behaviors, with the strongest effects observed in younger populations (eg, children and young adults) [10]. The review identified the need for more research into long-term effects (eg, beyond 12 months), specific health domains (eg, mental health), and optimal methods for supporting goal setting. A systematic literature review of 26 studies found that users of digital mental health technologies valued features that supported them in setting and tracking their progress with goals [11], and evidence suggests a link between goal achievement and increased user engagement. For example, a study with more than 4000 users aged 11 to 25 years on a digital mental health app showed that more than half (55.6%) of the users had meaningful improvement in the goals they set collaboratively with a practitioner [12]. Those who had meaningful improvement in their goals spent more time with the app than those who did not, suggesting goal achievement may be associated with higher engagement. Notably, the study only evaluated goals set collaboratively with a practitioner and did not examine relationships with user-set goals. Another study examining goal setting in adult users aged 18 years and older found that those with a practitioner-recorded presenting issue made the most progress in their goals, and goals were more frequently achieved when set with a practitioner than when set by users themselves [13]. Further research is needed to explore how both user-set and practitioner-set goals influence user engagement and psychological distress among adolescents and young adults.

The purpose of this paper was to examine the relationship between various goal-related variables on user engagement and mental health (ie, psychological distress) in a free digital mental health platform designed for adolescents and young adults. A secondary, exploratory aim was to assess how different user-presenting issues were associated with engagement. We hypothesized the following:

1. The number of goals created will be positively associated with increased engagement.
2. Progress in both user-set and practitioner-set goals will be positively associated with engagement.
3. Progress in user-set and practitioner-set goals will be associated with decreases in psychological distress.

## Methods

### Digital Mental Health Platform

Kooth is a free, innovative web-based mental health platform designed for children and young people aged 10 to 25 years in the United Kingdom. Children and young people hear about Kooth predominantly through schools, health care professionals, friends and family, community events, social media, or online search engines. For more than 2 decades, Kooth has provided the UK National Health Service with an anonymous mental health support platform that is accessible without a formal referral, provided Kooth is commissioned in their local area. Kooth provides a wide array of resources and support options, including therapeutic content, peer-created therapeutic articles, online community forums, asynchronous and synchronous messaging with practitioners, goal setting, emotional journaling, and a variety of self-guided well-being activities.

### Participants

Participants in this study were youth who engaged in Kooth and provided consent for their data to be used for research purposes during Kooth registration. Users could opt in or out for their platform data to be used for research purposes at any point by messaging the Kooth team inbox on the platform. All data were collected by Kooth as part of standard care practices. Data were included in analyses for this paper only for users who had data for goal-related variables (because not all users engaged with this feature on the autonomous platform) and completed a psychological distress measure before and after engaging with the goal feature. Only anonymized data that fit these criteria were shared with the researchers.

### Measures

#### Goals

##### Goal Creation

Users can set their own goals through the platform using the goal feature, independently of any practitioner interactions. A brief introduction to goals and how to monitor their goal is provided. These goals are moderated by the Kooth moderation team to check any risk related to safeguarding concerns. If a goal is not suitable or a risk is flagged, a practitioner will contact the user through the platform to discuss and safeguard, if needed, based on safeguarding processes in Kooth. Practitioners can also set goals collaboratively with users during chats. For analysis, goal creation was operationalized as the number of goals created, separated into user-set goals and practitioner-set goals. Both were treated as continuous count variables.

##### Goal Progress

The goal-based outcome tool is a widely used measure for evaluating progress toward goals in mental health interventions for children and young people [14]. This tool supports collaborative goal setting by involving the young person and, optionally, their practitioner. Goals can be established either by the young person, the practitioner, or both and are rated at regular intervals on an 11-point scale from 0 (no progress) to 10 (goal achieved). The goal-based outcome tool helps track progress by comparing initial goal settings with subsequent ratings, providing a clear measure of advancement over time, as all goals start from 0 by default. Goals are regularly reviewed and discussed with the practitioner for users who choose to attend chats on the platform. A movement of 3 points is considered a meaningful change, with higher scores reflecting more significant progress toward goal achievement [12].

For our analysis, we computed goal progress as the difference between the most recent follow-up score and the baseline score of 0 on the primary goal. This change score, which ranges from 0 to 10, reflects the amount of progress made; higher scores indicate greater improvement toward the goal.

##### Engagement

Platform engagement was operationalized as the number of logins recorded for each user between registration (start date) and last activity (end date). This was treated as a continuous variable in regression analyses. To provide additional descriptive context, we also reported the span of activity in days (number of days between registration and last active date), though this variable was not used in regression models.

##### Psychological Distress

Psychological distress was assessed via either the Young Person’s Clinical Outcomes in Routine Evaluation (YP-CORE; for users aged ≤16 years [15]) or Clinical Outcomes in Routine Evaluation-10 (CORE-10; for users aged ≥17 years [16]) at 6 time points. The final YP-CORE and CORE scores used in analyses represented the last registered score a user completed on the platform, which did not always correspond to their final time point of platform use. Both measures are 10 items that ask participants to rate their experiences of the past week on a 4-point Likert scale, ranging from “not at all” to “all or most of the time.” Example items include “I have felt despairing or hopeless” and “I have felt tense, anxious, or nervous.” Total scores range from 0 to 40, with higher scores indicating greater levels of psychological distress. These scales were used at registration to provide a baseline score of psychological distress. In chats, practitioners also used this scale to track and monitor psychological distress. Completion of these measures was always optional for users.

##### Presenting Issues

Presenting issues were psychological, social, or behavioral concerns disclosed by users during interactions on the platform, identified through one-to-one chats, moderation of journals or goal entries on the platform, or moderation of community engagement activities. Presenting issues were not mutually exclusive, meaning users could have multiple presenting issues. These concerns were systematically categorized based on their nature or impact. In this paper, categories included emotional difficulties; home and relationships; mental health; risk, abuse, or safeguarding risk; physical health; school-related issues; suicidality, self-harm, and maladaptive coping; and other presenting issues. The grouping of these presenting issues into these categories was validated by Kooth clinicians and researchers who provided the data extract. For analysis, each category was coded as a binary variable (0=not present and 1=present), and users could be represented in multiple categories simultaneously.

##### Demographic Data

Demographic data were routinely collected upon registration to the Kooth platform using predefined drop-down boxes where users selected their responses. Categories included age (measured in years), gender identity (female, male, gender fluid, agender, nonbinary, other self-disclosed, and prefer not to say), and ethnicity (White British, African, Asian, Black, mixed backgrounds, Bangladeshi, Caribbean, Chinese, Indian, Irish, Pakistani, any other White background, and not stated). Racial and ethnic categories were grouped as follows: White British, any other White background, African, not stated, and a combined “remaining ethnicities” category (including Asian, Black, mixed backgrounds, Bangladeshi, Caribbean, Chinese, Indian, Irish, Pakistani, White and Asian, White and Black African, and White and Black Caribbean, collectively representing 12.74% (88/691) of the sample, with each subroup individually accounting for fewer than 2% of participants.

### Statistical Analysis

Descriptive statistics were used to examine participant demographics (age, gender identity, and ethnicity), engagement, psychological distress, goals set by both the user and the practitioner (average number of goals set and goal progress), and presenting issues. Pearson correlations were used to assess relationships between these key variables.

Paired samples 2-tailed *t* tests were conducted to assess changes in psychological distress. Normality of difference scores was assessed using Shapiro-Wilk (W=0.932; *P*<.001) and Kolmogorov-Smirnov (D=0.173; *P*<.001) tests. Although both were significant, skewness (–0.683) and kurtosis (1.440) indicated only mild deviations, and Q-Q plots supported approximate normality. Given the large sample size (N=691), use of the paired *t* test was deemed robust to these mild deviations. These tests compared distress levels at registration and the user’s last distress score to determine whether significant changes occurred. Analyses controlled for age, ethnicity, and gender identity.

All regression models included relevant control variables (eg, age, gender identity, and ethnicity) to adjust for potential confounding factors. For predicting engagement, linear regression was used to examine how goal-related variables (number of goals created and goal progress) predicted user engagement. We ran three models, namely (1) user-set goal variables only, (2) practitioner-set goal variables only, and (3) both user-set and practitioner-set goal variables together, to explore their unique and combined contributions to engagement. An additional, separate linear regression was conducted to assess how user-presenting issues were related to user engagement. Presenting issues were analyzed in a separate regression to assess their relationship with platform engagement to maintain conceptual clarity and avoid model overfitting.

For predicting psychological distress, linear regressions were used to determine how goal-related variables (number of goals created and goal progress by the user and practitioner) predicted decreases in psychological distress. Specifically, we first conducted a regression model, including both user-set and practitioner-set goals variables (number of goals created and progress) as simultaneous predictors, to assess their unique contributions while accounting for shared variance. We then conducted separate regression models for user-set and practitioner-set goals, each including both the number of goals created and goal progress, to examine their individual associations with changes in distress scores.

### Ethical Considerations

This study is a retrospective analysis of existing Kooth platform data. At registration, users choose whether their anonymized data may be used for research, and they can change this preference at any time. Only data from users who opted in were included. All data were fully anonymized prior to analysis, and no compensation was provided, as the data reflect naturally occurring platform use. The project design was reviewed and approved by the university research ethics committee at the University of Manchester, United Kingdom (2024-20916-36703).

## Results

### Overview

The study sample for this paper included 691 Kooth users in the United Kingdom who registered on the platform between January 3, 2020, and December 14, 2023. Notably, a larger number of users engaged with the platform over this time frame, but this analysis focused solely on those who provided consent to participate in the research and met the criteria for this study (ie, setting a goal and having psychological distress scores recorded both before and after setting a goal; a high proportion of users did not have a second psychological distress score). Participants logged in an average of 262.5 times (SD 520.84; range 1-7179 logins). The average interval between participants’ registration date and last recorded activity was 98.15 (SD 122.25; median 57; IQR 11-132) days, with a range of 1 to 1134 days, reflecting varied use over time by young people as needed. The average number of user-set goals in the entire sample was very low (<1 goal), largely due to a lack of data for user-set goals, indicating that users were choosing not to engage with the goals feature independently. However, among users who set at least 1 goal themselves, users set an average of 3 goals. For goals set collaboratively with a practitioner in the entire sample, an average of 9 goals were set. Among users whose practitioners set at least 1 goal with them, an average of 13 goals were set. The lack of data for user-set goals also impacted the sample size for goal progress, with user-set goal progress (n=116) having a lower sample size compared to practitioner-set goal progress (N=691).

Participants had a mean age of 15.84 (SD 2.88) years at registration, with ages ranging from 10 to 25 years. Among the 691 participants, the gender identity distribution was predominantly female (n=522, 75.54%), with smaller percentages self-identifying as male (n=112, 16.21%), gender fluid (n=22, 3.18%), agender (n=17, 2.46%), nonbinary (n=8, 1.16%), and other identities (self-disclosed: n=2, 0.28%; preferred not to say: n=8, 1.16%). Participants were predominantly White British (n=544, 78.72%) followed by those from another White background (n=26, 3.76%), African (n=17, 2.46%), not stated (n=16, 2.32%), and each of the remaining ethnicities were all less than 2%, collectively representing 12.74% (88/691) of the population (Bangladeshi, Caribbean, Chinese, Indian, Irish, Pakistani, White and Asian, White and Black African, White and Black Caribbean, and any other Asian, Black, Mixed, or another ethnic group background). Table 1 presents all participant descriptive statistics.

**Table 1:** Descriptive statistics (N=691).

Demographics		Values
**Gender identity, n (%)**		
	Female	522 (75.54)
	Male	112 (16.21)
	Gender fluid	22 (3.18)
	Agender	17 (2.46)
	Prefer not to say	8 (1.16)
	Nonbinary	8 (1.16)
	Other self-disclosed	2 (0.28)
**Race and ethnicity, n (%)**		
	White British	544 (78.72)
	Any other White background	26 (3.76)
	African	17 (2.46)
	Not stated	16 (2.32)
	Remaining ethnicities^a^	88 (12.74)
Age at registration (y), mean (SD)		15.84 (2.88)
**Goal variables, mean (SD)**		
	Number of user-set goals	<1 (1.88)
	User-set goal progress (0-10)	5.69 (4.04)
	Number of practitioner-set goals	9.04 (10.47)
	Practitioner-set goal progress (0-10)	4.68 (3.40)
Platform engagement^b^ (logins), mean (SD)		262.5 (520.84)
**Psychological distress (0-40), mean (SD)**		
	Preengagement	27.15 (7.10)
	Postengagement	26.16 (8.27)
	Difference	–2.47 (6.93)
**Presenting issues^c^ (yes or no), n (%)**		
	Suicidality, self-harm, or maladaptive coping	333 (48.19)
	Emotion issue	330 (47.75)
	Home or relationship issues	345 (49.92)
	Mental health issue	391 (56.58)
	Other issue	117 (16.93)
	Risk, abuse, or safeguarding risk issue	194 (28.07)
	Physical issue	116 (16.78)
	School issue	257 (37.19)

^a^“Remaining ethnicities” included any other Asian, Black, mixed backgrounds, Bangladeshi, Caribbean, Chinese, Indian, Irish, Pakistani, White and Asian, White and Black African, and White and Black Caribbean.

^b^Data for the engagement variable were from the platform between January 3, 2020, and December 14, 2023, and did not reflect overall platform engagement for all users.

^c^Presenting issues were not mutually exclusive; users may have presented with any combination of issues or none at all.

### Engagement Predictors

Linear regression analysis revealed that goal progress, whether for self-set (*P*=.56) or practitioner-set goals (*P*=.30), and the number of self-set goals (*P*=.18) were not associated with engagement. However, the number of practitioner-set goals was positively associated with engagement (β=.16; *P*<.001). Four types of presenting issues were significantly positively associated with increased engagement (Table 2), including physical issues (β=.21; *P*<.001); school issues (β=.17; *P*<.001); risk, abuse, or safeguarding issues (β=.14; *P*=.002); and other issues (β=.10; *P*=.01).

**Table 2:** Linear regression results of the user presenting issues and the Kooth platform engagement.

Independent variable			B^a^ (SE; 95% CI)		β^b^		*P* value
Suicidality, self-harm, or maladaptive coping			48.64 (51.75; –52.98 to 150.26)		.04		.35
Emotion issue			58.26 (60.30; –90.16 to 176.67)		.06		.33
Home or relationship issues			16.49 (60.95; –103.20 to 136.28)		.02		.79
Mental health issue			–102.95 (63.77; –228.17 to 22.28)		–.09		.11
Other issue			94.76 (56.71; –16.60 to 206.12)		.07		.09
Risk, abuse, or safeguarding risk issue			163.38 (52.93; 59.45 to 267.31)		.14		.002
Physical issue			294.99 (56.65; 183.74 to 406.24)		.21		<.001
School issue			182.00 (52.96; 78.02 to 285.986)		.17		<.001
**Controls**							
	Age	2.41 (6.19; –9.73 to 14.57)		.01		.70	
	Ethnicity^c^	71.64 (47.78; –22.18 to 165.47)		.05		.13	
	Agender^d^	–9.68 (118.71; –242.78 to 223.42)		–.03		.94	
	Gender fluid^d^	–118.24 (107.48; –329.28 to 92.81)		–.04		.27	
	Nonbinary^d^	–108.14 (173.16; –448.16 to 231.87)		–.02		.53	
	Other self-disclosed^d^	–236.11 (342.89; –909.41 to 437.20)		–.02		.49	
	Male^d^	–100.95 (51.16; –201.41 to –0.49)		–.07		.049	

^a^Unstandardized regression coefficient. SEs and CIs apply to B only.

^b^Standardized coefficient.

^c^Reference group: not British.

^d^Reference group: female.

### Psychological Distress

Among youth aged 10 to 16 years, 87.44% (390/446) scored above the established clinical cutoff for significant psychological distress on the YP-CORE. For young adults aged 17 years and older, 60% (147/245) experienced severe distress on the CORE-10, 20% (49/245) experienced moderate to severe distress, and 13.06% (32/245) experienced moderate distress, with the remainder experiencing mild, low, or healthy distress levels.

### Changes in Psychological Distress Over Time

The paired samples *t* test showed a significant decrease in psychological distress from time point 1 to time point 2 across all users (t_683_=–9.32; *P*<.001). The mean difference was –2.47 (SD 6.93) points, indicating a small to medium effect size (Cohen *d*=–0.356). When examining differences by age (10-16 y and ≥17 y), both groups showed significant decreases in psychological distress. For those aged 16 years and younger (t_445_=–6.97; *P*<.001), the mean difference was –2.35 and Cohen *d* was –0.330. For those aged 17 years and older (t_244_=–6.36; *P*<.001), the mean difference was –2.67 and Cohen *d* was –0.41. The effect size was slightly higher for the older group.

### Relationship Between Goal-Related Variables and Psychological Distress

Linear regression results indicated that greater progress in goals set with a practitioner was significantly associated with a decrease in psychological distress (β=–.27; t_563_=–6.360; *P*<.001) after controlling for engagement. Specifically, for each 1-unit increase in goal progress, psychological distress decreased by 0.59 units (β =–.59, 95% CI –0.77 to –0.41).

Linear regression results indicated that greater progress in goals set by the user themselves was not significantly associated with changes in psychological distress (β=–.14; t_103_=–1.405; *P*=.16), after controlling for engagement. Results from linear regression analyses are presented in Table 3.

**Table 3:** Linear regression results of practitioner-set goal progress and the user’s change in psychological distress.

Independent variable		B^a^ (SE; 95% CI)	β^b^	*P* value
Practitioner-set goal progress		–.579 (0.09; –0.77 to –0.40)	–.27	<.001
**Controls**				
	Age	0.06 (0.10; –0.14 to 0.25)	.02	.58
	Ethnicity^c^	1.74 (0.79; 0.19 to 3.28)	.09	.03
	Agender^d^	1.24 (1.89; –2.46 to 4.95)	.03	.51
	Gender fluid^d^	–2.13 (1.67; –5.45 to 1.19)	–.05	.21
	Nonbinary^d^	4.66 (2.95; –1.14 to 10.46)	.06	.12
	Other self-disclosed^d^	–0.82 (5.10; –10.85 to 9.20)	–.01	.87
	Male^d^	–1.65 (0.91; –3.44 to 0.14)	–.07	.07
	Engagement (d)	0.001 (0.001; 0.00 to 0.002)	.09	.03

^a^Unstandardized regression coefficient. SEs and CIs apply to B only.

^b^Standardized coefficient.

^c^Reference group: not British.

^d^Reference group: female.

## Discussion

### Principal Findings

The purpose of this paper was to examine the relationship among various goal-related variables, presenting issues, engagement, and mental health (ie, psychological distress) in a free digital mental health platform designed for adolescents and young adults. This platform provides an observational perspective on how youth choose to autonomously engage with the platform and goal setting, both by setting goals themselves and collaboratively with a practitioner. Our primary aim was to understand how goal setting and achievement are related to platform engagement and changes in psychological distress among adolescents and young adults using a digital mental health platform. We also explored how different users’ presenting issues were related to platform engagement. Our findings revealed that the number of practitioner-set goals was associated with platform engagement, whereas other goal-related variables were not associated with platform engagement. In addition, presenting issues related to physical health; school; and risk, abuse, or safeguarding were associated with platform engagement. Progress in collaboratively set goals by practitioners was linked to a decrease in psychological distress.

Within this sample, the number of practitioner-set (but not self-set) goals created was positively associated with user engagement, while goal progress was not. This suggests that practitioner involvement in collaboratively setting goals with users may play an important role in fostering engagement, aligning with broader evidence from digital mental health research showing that human contact is a key factor in sustaining platform engagement [17]. It is possible that the relationship operates in the other direction—that more engaged users are also more likely to connect with practitioners and set goals collaboratively. In this context, practitioner-set goals may reflect engagement rather than drive it, whereas self-set goals may lack the accountability and support needed to sustain platform use. Other potential influences on engagement, such as peer support features or users’ personal motivations, may influence engagement but were not captured in this study [18]. Notably, this dataset was restricted to focus specifically on goal-related variables from users who had 2 measurement points before and after goal engagement relating to psychological distress. This limited the sample and therefore does not capture all factors that may influence user engagement.

Interestingly, users presenting with physical health issues, school-related issues, and risk, abuse, or safeguarding issues demonstrated the strongest associations with engagement in the platform. This suggests that these specific presenting issues might be more salient or urgent for users, prompting users to engage more actively with Kooth. Given the platform’s primary advertisements through schools and communities, it may have resonated more with youth experiencing challenges related to school and physical health, particularly because the sample was collected during and shortly after the COVID-19 pandemic, when many youth faced ongoing school-related difficulties [19,20]. The nonsignificant relationship between engagement in Kooth and mental health and home or relationship issues suggests that these specific presenting concerns may not be the sole factors driving how young people engage with the platform. It is possible that users view Kooth as a broader support resource, using it to address a wide range of life challenges beyond mental health alone. This aligns with how Kooth is marketed in the United Kingdom, which emphasizes its role as an early intervention and prevention tool for higher-need populations, offering solution-focused support and encouraging users to seek additional mental health resources (eg, neurodivergence assessments or social care) [21,22]. Our findings are also consistent with previous research showing that users of the digital mental health platform, including youth and young adults, present with a wide range of issues (eg, self-worth, work-related challenges, and physical health concerns) [13,20]. In addition, one study found that users set goals in various areas, including self-help, self-care, emotional exploration, career aspirations, and self-help skills for life, demonstrating the platform’s focus on a wide range of personal development beyond mental health [13]. However, it is also possible that the lack of association between mental health and home or relationship issues with engagement in the platform reflects alternative coping strategies, greater reliance on offline support, or different help-seeking preferences among users presenting with these issues. Future research is needed to more fully understand the specific drivers of engagement and the diverse ways young people use Kooth.

Users’ psychological distress significantly decreased over time, indicating that the platform had a positive impact on mental health outcomes. However, the relationship between goal progress and decreases in psychological distress reduction was not the same across user-set goals and collaboratively set goals between users and a practitioner. Specifically, progress in practitioner-set goals was associated with a significant decrease in psychological distress, while progress in user-set goals did not show the same relationship (ie, nonsignificant). This finding suggests that goals set with trained practitioners may be more effective in reducing psychological distress than those set independently by users. Practitioner-set goals are likely to be more targeted, structured, and grounded in professional therapeutic approaches, which could explain their greater impact. These results align with existing research that highlights the importance of professional guidance in goal setting within mental health interventions [7,9]. However, the lack of a significant association between user-set goal progress and decreases in psychological distress might be attributable to users setting fewer goals on their own than with a practitioner, as the sample size for user-set goal progress (n=116) was substantially smaller than that for practitioner-set goal progress (N=691).

### Limitations

This study had several limitations. First, the small sample size for some variables restricted the ability to stratify results by demographic factors, such as age, gender identity, or ethnicity, which could have provided more detailed insights into different user groups. Second, the sample was predominantly female, which may limit generalizability. Third, the time frame was limited to the period when goals were set, which may not fully capture the long-term impact of engagement on the entire platform. Fourth, more granular use metrics (eg, feature use, session length, and frequency patterns) were not available, which limits the ability to capture the full range of user engagement behaviors. Fifth, presenting issues were determined by practitioners based on text-based chats rather than self-reports from users, which may introduce bias in how issues are categorized and understood. Finally, as the user’s choice is highly important to Kooth, users were not required to complete the psychological distress measures. Collecting these data outside of the routinely collected data would provide a more complete dataset, although leveraging real-world data is a strength, as it provides a more accurate understanding of the true effectiveness of digital platforms.

### Future Research

Future research with larger sample sizes should further investigate the impact of both self-set and practitioner-set goals on psychological distress to better understand how these different types of goals influence mental health outcomes. Larger samples would provide more robust data to examine potential differences in goal-setting mechanisms and their effectiveness. Future research should aim to include a more balanced gender identity distribution to better understand potential differences in outcomes across gender identities. In addition, this research could help identify whether self-set goals have a distinct or complementary effect compared to practitioner-set goals, ultimately informing more tailored and effective strategies for improving psychological distress. Future research could include a qualitative study to explore what practitioners are doing to set goals and whether this is a mechanism that improves psychological distress progress, particularly in scaling digital platforms. In addition, understanding how users can be guided to set their own goals outside of practitioner engagement would be valuable for developing tools that facilitate greater user autonomy. Considering users did not engage with the goals feature as much on their own compared to setting goals with a practitioner, one suggestion is to revise the platform’s language to guide users through the independent goal-setting process, making it more integrated into the platform journey to create a more guided experience similar to that of practitioner-set goals. Future studies should consider additional demographic factors (eg, socioeconomic status) or other individual characteristics to explore how these factors may influence the relationship between goal progress and psychological distress. In addition, examining these relationships in other populations beyond the United Kingdom would improve generalizability across young people. These recommendations are consistent with a recent scoping review where the need for more research to better define, implement, and measure goal-oriented practices in youth mental health was highlighted [23].

### Conclusions

This study examined the relationship among presenting issues, goal setting, and progression (both user set and those set collaboratively with a practitioner) and young people’s engagement with a free, anonymous digital mental health platform, Kooth. We also examined the impact of goal setting and progress on users’ psychological distress. We leveraged a unique dataset that provided real-world evidence from a nonstructured digital platform where users had the autonomy to engage with different aspects of the platform based on their preferences. Broadly, we found that progress in goals set collaboratively with a practitioner predicted a decrease in psychological distress. For users’ presenting issues, school-related and physical health concerns were the strongest predictors of platform engagement. The Kooth platform may have the potential to serve as a resource for a wide range of young people’s mental health needs, providing vital early intervention and prevention support for navigating diverse challenges, including those beyond mental health.

Importantly, this study highlights the value of setting and progressing toward goals, particularly those collaboratively established with practitioners. Goals cocreated with practitioners were associated with decreases in psychological distress while self-set goals were not, emphasizing the role of guided support in making meaningful changes in mental health. Fostering collaboration between young users and practitioners may optimize mental health improvements on digital mental health platforms. This paper adds to the existing literature by deepening our understanding of how goal setting and achievement can be optimized in digital mental health platforms to better support adolescents and young adults facing a variety of issues, while also providing insights into how engagement differs based on presenting issues.

## Abbreviations

CORE: Clinical Outcomes in Routine Evaluation
YP-CORE: Young Person’s Clinical Outcomes in Routine Evaluation

## Data Availability

The datasets presented in this study are not readily available because they are proprietary and subject to confidentiality restrictions. Requests to access the datasets should be directed to Kooth Digital Health (research@kooth.com).
